# Supplementary figures and images for: Characterization of the diversity of barn owl’s mitochondrial genome reveals high copy number variations in the control region
Source: PLoS One. 2024 Jan 25;19(1):e0295595. doi: 10.1371/journal.pone.0295595 (PMC10810427; doi:10.1371/journal.pone.0295595)

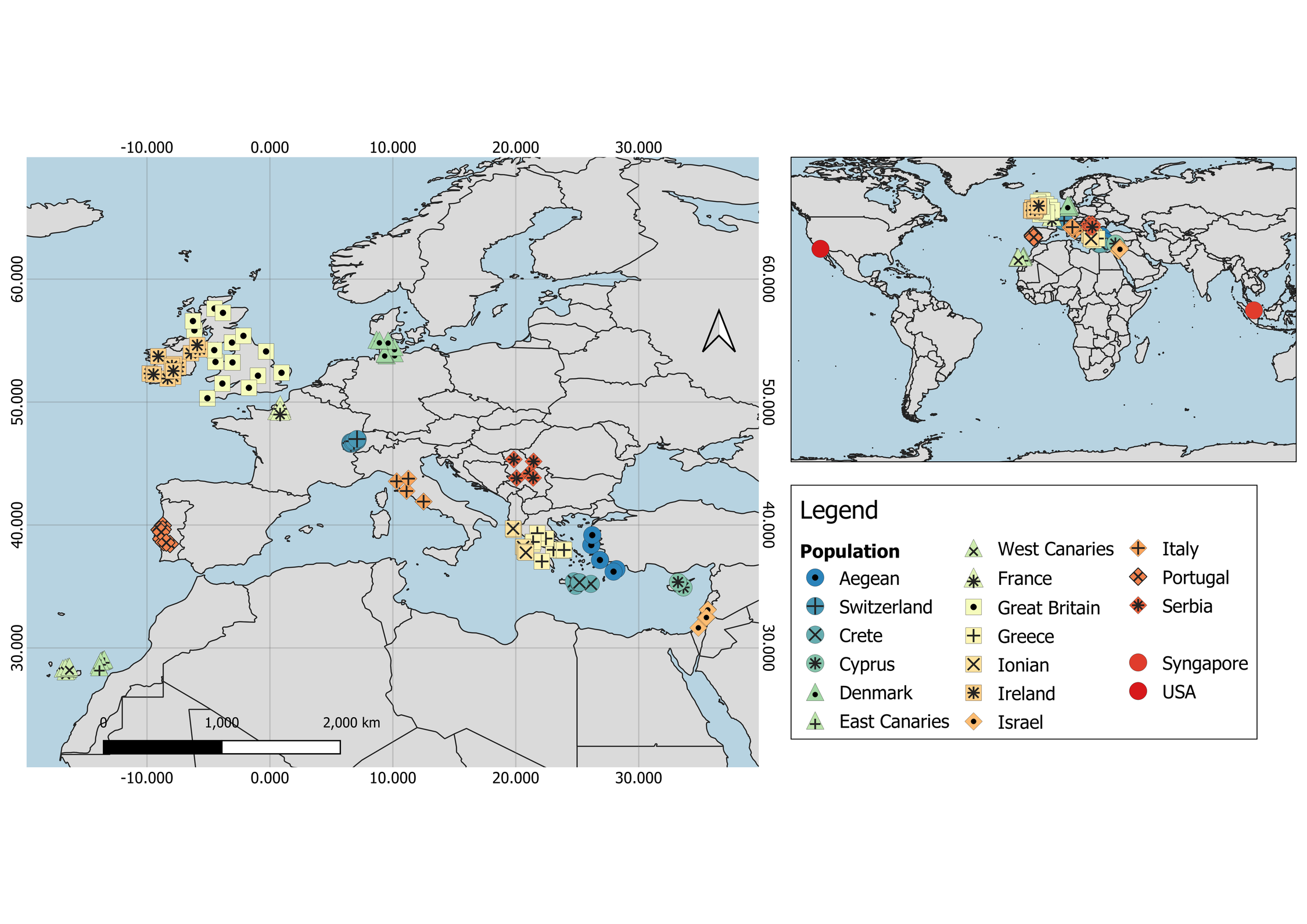

Supplement: S1 Fig — A. Individuals collected in Europe and its surroundings. B. Sampling locations including outgroups from USA and Singapore. Population symbols correspond to the ones shown in Fig 2. (TIF) [file pone.0295595.s005.tif]

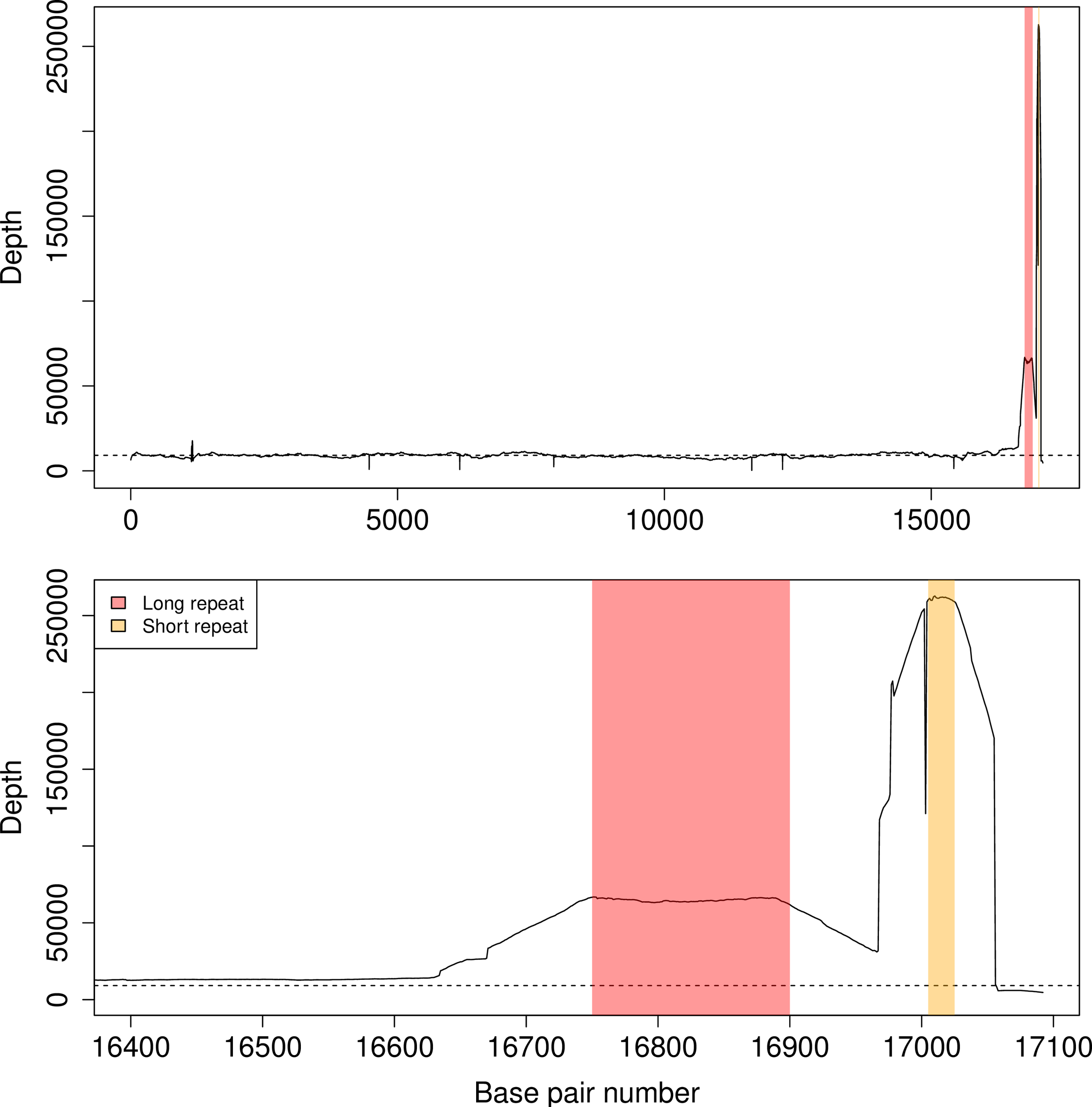

Supplement: S2 Fig — (A) Depth along the complete genome. (B) Close up on the non-coding region where the repeats are located. The vertical lines represent the delimitation of the plateau where the mean depth for the repeated regions. The horizontal straight line represents the median depth for all base pairs in the two panels. (TIF) [file pone.0295595.s006.tif]

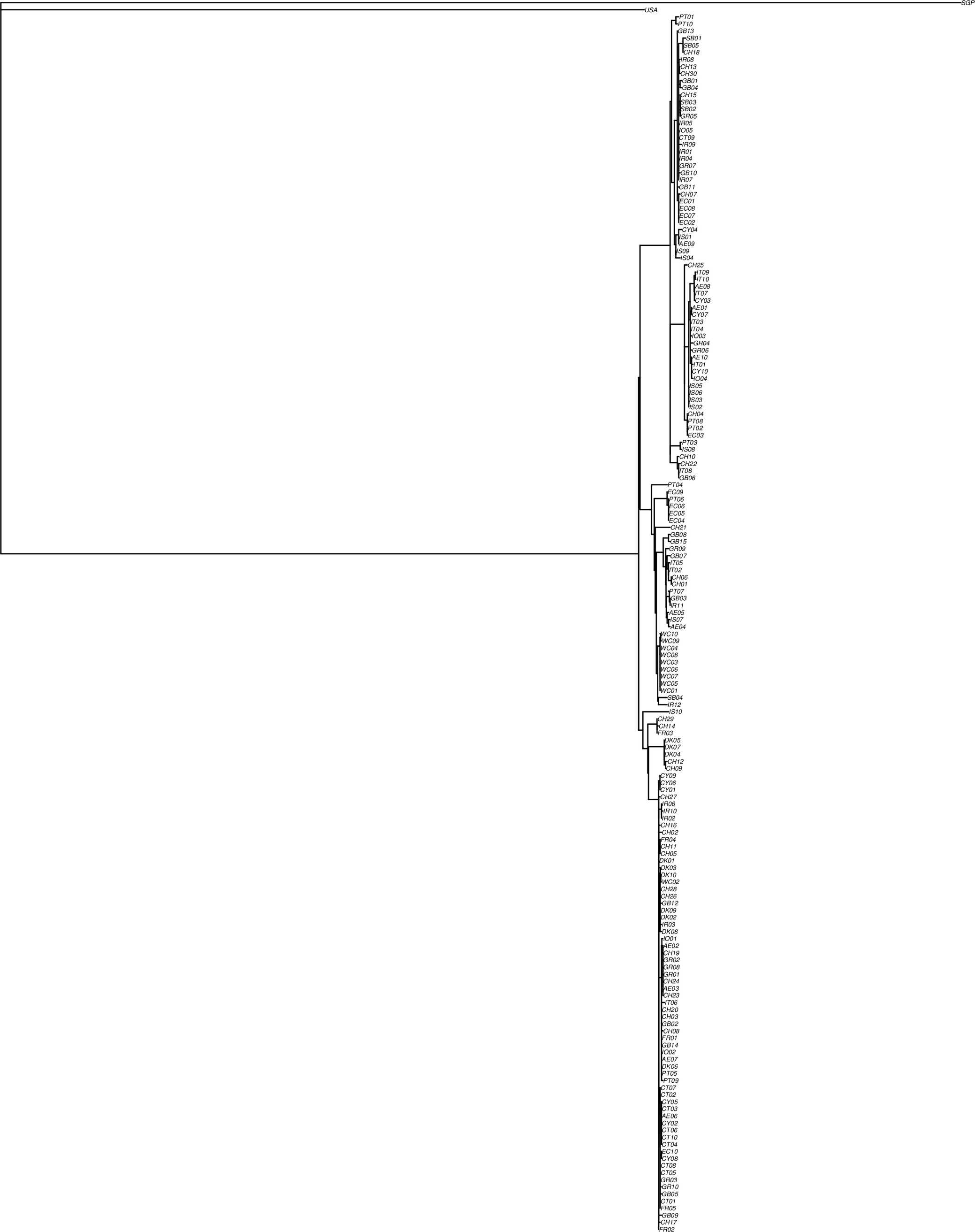

Supplement: S3 Fig — Branch lengths correspond to the genetic distance between individuals. (TIF) [file pone.0295595.s007.tif]

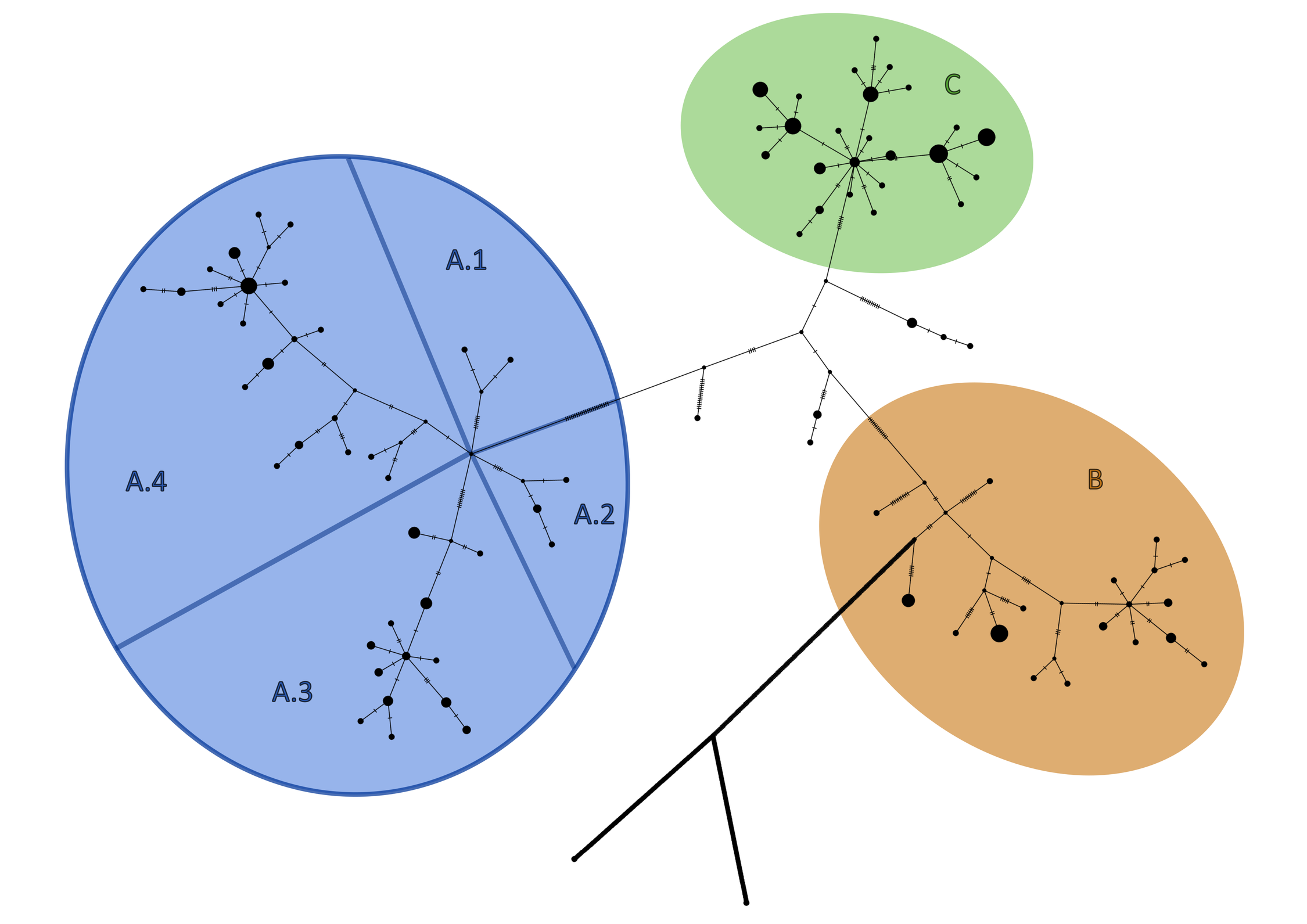

Supplement: S4 Fig — Different colors represent the haplogroups in which the individuals were clustered. The individuals that didn’t cluster clearly with any group were excluded from the analysis. USA and Singapore individuals were also discarded based on their high differentiation from the rest of the sample. Each tick on the network branches represents a mutation. (TIF) [file pone.0295595.s008.tif]

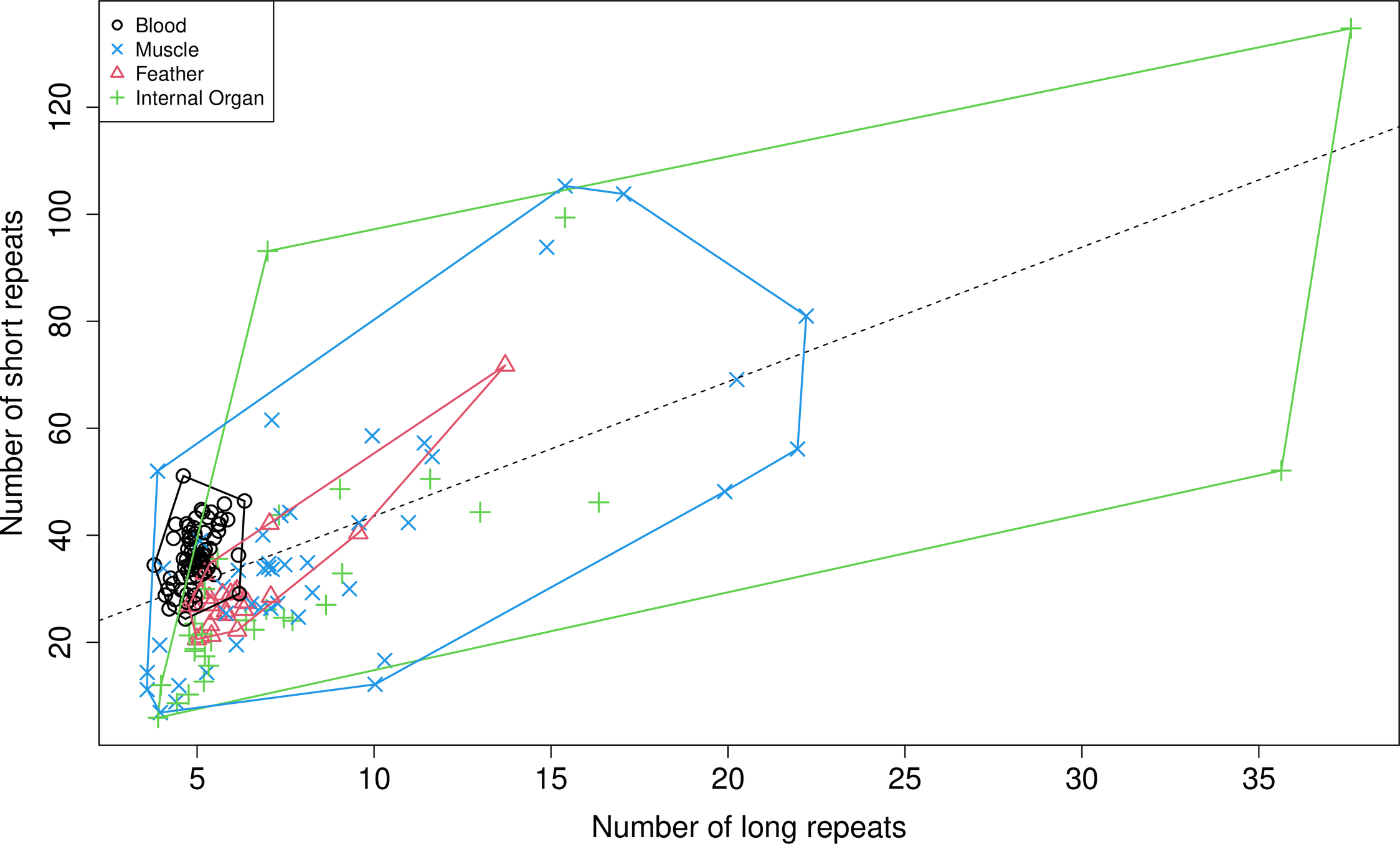

Supplement: S5 Fig — Convex hulls were drawn to show the distribution of number of repeats between the different tissues. There is a strong correlation between both numbers of repeats r = 0.65, r2 = 0.42, p = 2x10^-16^. (TIF) [file pone.0295595.s009.tif]

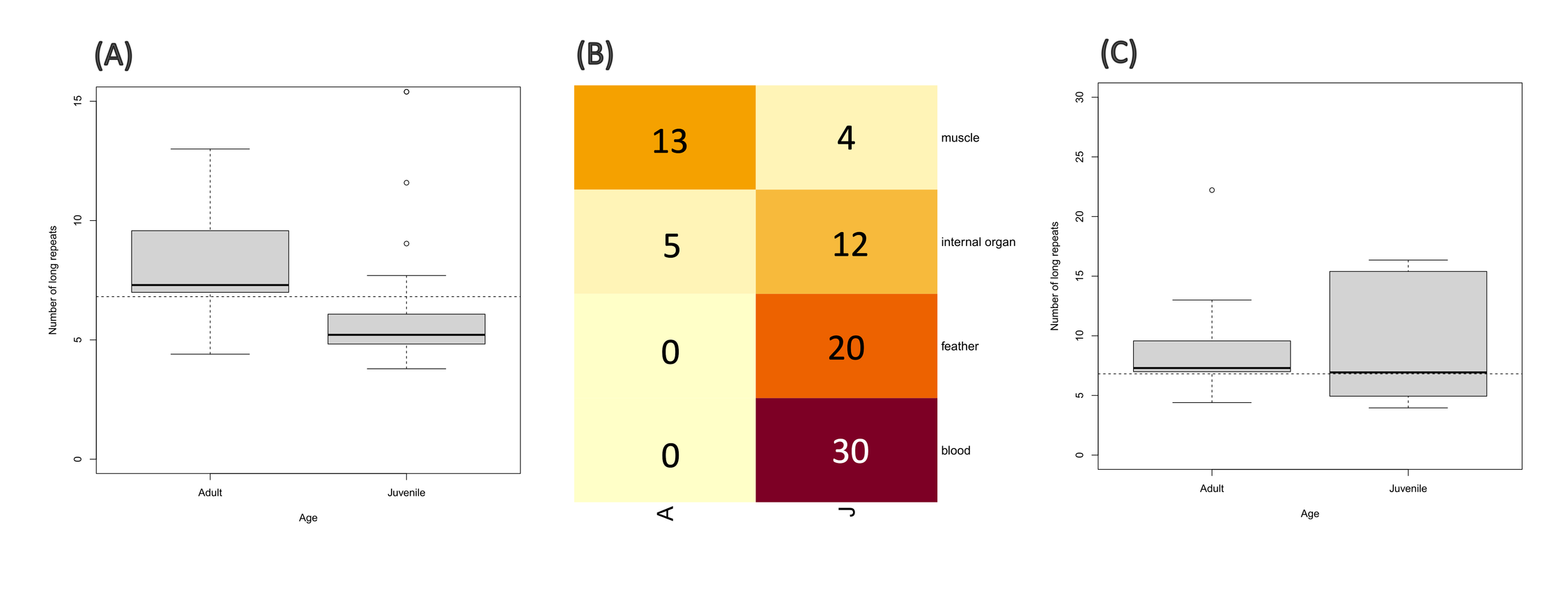

Supplement: S6 Fig — (A) The number of repeats using age as the explanatory variable. (B) Heatmap of the contingency table of age in regard to tissue. Higher number of individuals corresponds to a higher color intensity. (C) The number of repeats using age as the explanatory factor while using only individuals that were collected form muscle or internal organs. (TIF) [file pone.0295595.s010.tif]

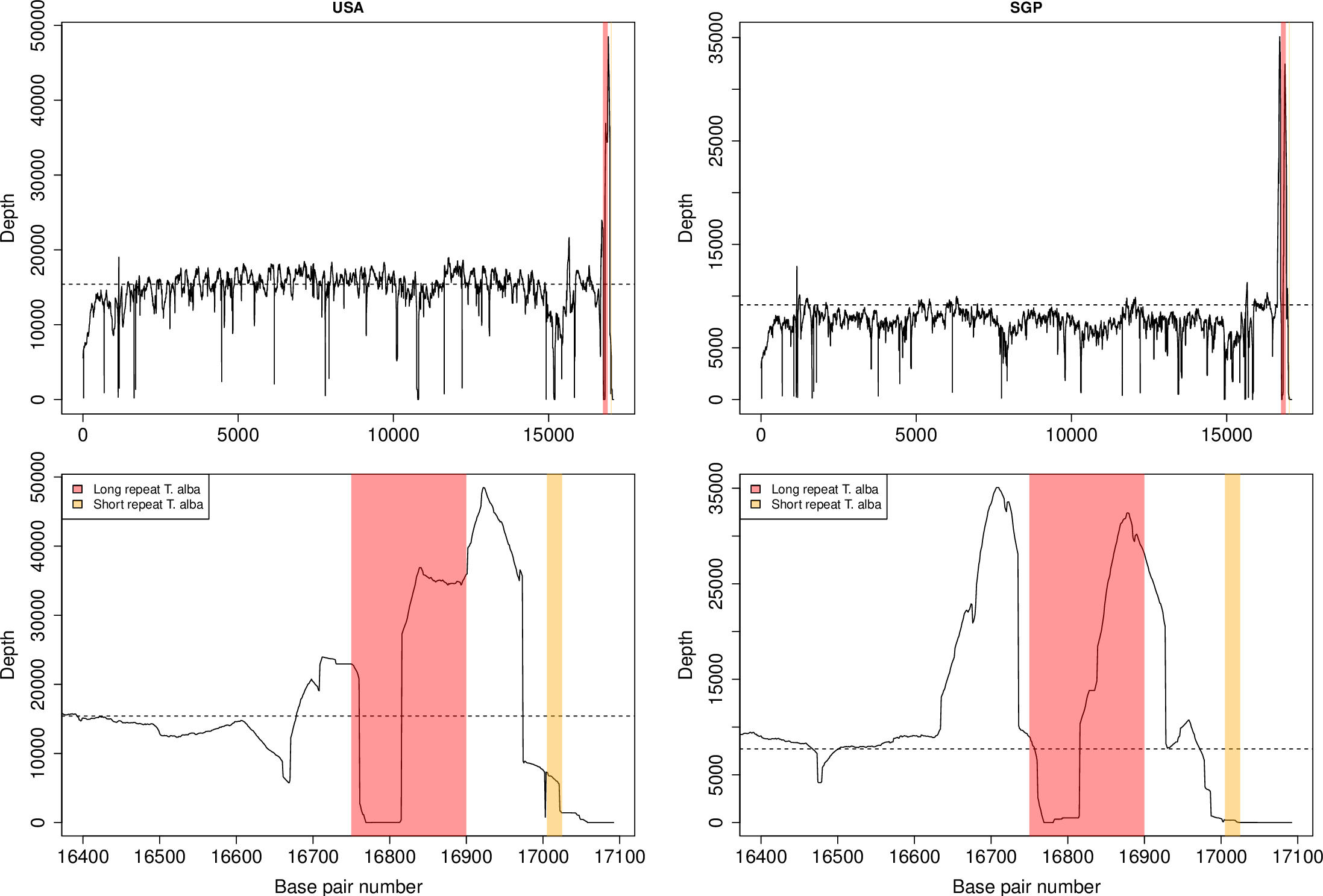

Supplement: S7 Fig — (A) Depth along the complete genome. (B) Close up on the non-coding region where the repeats are located in T. alba. The horizontal straight line represents the median depth for all base pairs in the two panels. (TIF) [file pone.0295595.s011.tif]
