# Supplementary material for: Characterization of the diversity of barn owl’s mitochondrial genome reveals high copy number variations in the control region
Source: PLoS One. 2024 Jan 25;19(1):e0295595. doi: 10.1371/journal.pone.0295595 (PMC10810427; doi:10.1371/journal.pone.0295595)
Supplement: Attachment — Submitted filename: 0_Tyto_mtDNA_AnswerReviewer.docx [file pone.0295595.s012.docx]

PONE-D-23-20889

Characterization of the diversity of barn owl’s mitochondrial genome reveals high copy number variations in the control region.

PLOS ONE

-------------------------------------------------------------------------------------------------------------------------------------------

Journal Requirements:

When submitting your revision, we need you to address these additional requirements.

1. Please ensure that your manuscript meets PLOS ONE's style requirements, including those for file naming. The PLOS ONE style templates can be found at

https://journals.plos.org/plosone/s/file?id=wjVg/PLOSOne_formatting_sample_main_body.pdf and

https://journals.plos.org/plosone/s/file?id=ba62/PLOSOne_formatting_sample_title_authors_affiliations.pdf

[R] We have now formatted our manuscript according to style requirements.

2. Please note that funding information should not appear in any section or other areas of your manuscript. We will only publish funding information present in the Funding Statement section of the online submission form. Please remove any funding-related text from the manuscript.

[R] We removed this sentence in the new version of the manuscript.

3. Thank you for stating the following financial disclosure:

"This work was supported by the Swiss National Science Foundation (https://www.snf.ch/en) with grants 31003A_179358 & 310030_215709 to JG."

Please state what role the funders took in the study. If the funders had no role, please state: "The funders had no role in study design, data collection and analysis, decision to publish, or preparation of the manuscript."

If this statement is not correct you must amend it as needed.

Please include this amended Role of Funder statement in your cover letter; we will change the online submission form on your behalf.

[R] We confirm that: "The funders had no role in study design, data collection and analysis, decision to publish, or preparation of the manuscript."

4. Please include captions for your Supporting Information files at the end of your manuscript, and update any in-text citations to match accordingly. Please see our Supporting Information guidelines for more information: http://journals.plos.org/plosone/s/supporting-information.

[R] The new version of the manuscript includes the caption of the Supporting Information.

5. We note that supplementary Figure 1 in your submission contain map/satellite images which may be copyrighted. All PLOS content is published under the Creative Commons Attribution License (CC BY 4.0), which means that the manuscript, images, and Supporting Information files will be freely available online, and any third party is permitted to access, download, copy, distribute, and use these materials in any way, even commercially, with proper attribution. For these reasons, we cannot publish previously copyrighted maps or satellite images created using proprietary data, such as Google software (Google Maps, Street View, and Earth). For more information, see our copyright guidelines: http://journals.plos.org/plosone/s/licenses-and-copyright.

We require you to either (a) present written permission from the copyright holder to publish these figures specifically under the CC BY 4.0 license, or (b) remove the figures from your submission:

a. You may seek permission from the original copyright holder of supplementary Figure 1 to publish the content specifically under the CC BY 4.0 license.

We recommend that you contact the original copyright holder with the Content Permission Form (http://journals.plos.org/plosone/s/file?id=7c09/content-permission-form.pdf) and the following text:

“I request permission for the open-access journal PLOS ONE to publish XXX under the Creative Commons Attribution License (CCAL) CC BY 4.0 (http://creativecommons.org/licenses/by/4.0/). Please be aware that this license allows unrestricted use and distribution, even commercially, by third parties. Please reply and provide explicit written permission to publish XXX under a CC BY license and complete the attached form.”

Please upload the completed Content Permission Form or other proof of granted permissions as an "Other" file with your submission.

In the figure caption of the copyrighted figure, please include the following text: “Reprinted from [ref] under a CC BY license, with permission from [name of publisher], original copyright [original copyright year].”

b. If you are unable to obtain permission from the original copyright holder to publish these figures under the CC BY 4.0 license or if the copyright holder’s requirements are incompatible with the CC BY 4.0 license, please either i) remove the figure or ii) supply a replacement figure that complies with the CC BY 4.0 license. Please check copyright information on all replacement figures and update the figure caption with source information. If applicable, please specify in the figure caption text when a figure is similar but not identical to the original image and is therefore for illustrative purposes only.

The following resources for replacing copyrighted map figures may be helpful:

USGS National Map Viewer (public domain): http://viewer.nationalmap.gov/viewer/

The Gateway to Astronaut Photography of Earth (public domain): http://eol.jsc.nasa.gov/sseop/clickmap/

Maps at the CIA (public domain): https://www.cia.gov/library/publications/the-world-factbook/index.html and https://www.cia.gov/library/publications/cia-maps-publications/index.html

NASA Earth Observatory (public domain): http://earthobservatory.nasa.gov/

Landsat: http://landsat.visibleearth.nasa.gov/

USGS EROS (Earth Resources Observatory and Science (EROS) Center) (public domain): http://eros.usgs.gov/#

Natural Earth (public domain): http://www.naturalearthdata.com/

[R] We have changed the supplementary Figure 1. It now uses the maps from Natural Earth (public domain) and should be suitable for publication.

--------------------------------------------------------------------------------------------------------------------------------------------

Additional Editor Comments

One of the reviewers raised important concerns about study design and the drawn conclusions of the manuscript and I fully agree with these concerns. Therefore, I would suggest that the study design and questions studies are carefully reconsidered and the necessary revisions made before resubmission.

[R] We have addressed all the comments raided by the reviewers. Please find bellow our point-by-point answers.

--------------------------------------------------------------------------------------------------------------------------------------------

Review Comments to the Author

Reviewer #1: I think this article would be suitable for publication. This article could be useful for conservation policy of endangered owls later on. I hope the authors need to continue more investigation of endangered owl for the future.

[R] We are thankful of the referee for his/her positive feedbacks on our work.

Reviewer #2: The manuscript submitted by Serrano et al. describes substantial length variation observed in the control region of Tyto alba. The authors used a previously mitochondrial genome they obtained (Machado et al. 2022, MZ318036.1) that they here annotated. On genbank, the mtdna genome was specified to have rather low coverage (10X). The annotation provided by the authors would suggest very unusual features (in face unique to bird to my knowledge and i have checked or sequenced litterally hundred of complete mt dna genomes), which includes 5 independent duplication of five coding loci (ND2, CO1, ATP8, ND3, ND5) ! I then checked the Suppl. Table 1 and realised that it is not duplication but more likely wrong automatic annotation (it is actually a bit embarassing for the authors to not have checked this). The authors may perform manual curation of the annotation and compare it with closely related species for start end end of loci ; for example ND2, if merging _a and _b would be 1047 bp which is plausible (usually it is 1041 bp but a 2 codon insertion is possible).

[R] We thank the reviewer for pointing to this issue with the automatic annotation. As we mentioned in our previous version of the manuscript, we did not made the differentiation between spited annotation and duplicated genes (“Split or duplicated genes included […]”).

We have now manually curated the annotation, and all the genes mentioned were indeed spited in the automatic annotation. We have now changed the annotation and modified the main text and figure 1 accordingly. See figure 1 and line 88:

“We annotated the genome using Mitos Web Server (16) and manually curated the annotation.”

We have also removed the sentence mentioned above about spited and duplicated genes, see line 179.

Remaining analyses appear to be well done but i have a few questions regarding sequencing biases and how it would impact conclusions regarding the impact of age/island, etc

I actually wonder if there could not be across individual variation in the number of sequenced reads and thus inference in the number of repeats ? Could not we figure that the sequencing (hence coverage) of repeated regions can be more variable (with higher variance) than less repeated regions ? That tissue type would be linked to sequencing biases rather than number of copies per se ? What about numts numts ? How is overall coverage (feather versus muscle versus blood) ? For organ samples, could there be a bias linked to sample preservation ? for example, blood or feather will be preserved immediatly whereas it is likely that for organs there would be time between death and freeze or death and sampling and potentially different freeze/thaw cycles and thus sample degradation. All of the hypotheses tested by the authors are very interesting but i wonder what could actually be said with. I think the authors have something very interesting to go after but as they noted, they can not properly test most factors so i am unsure what could be concluded. For this purpose a careful sampled design may be needed to forrmely test their hypotheses.

[R] We agree with the reviewer that multiple factors can bias our results.

Regarding the tissue used, this is something we mentioned in our previous version. We have now added a caution note about the way tissue were conserved. see line 306:
“The individuals were sampled over a large time span and in different locations, resulting in different sampling procedures between them. Due to this non homogeneity of the sampling procedure, the samples cover different ages or sexes. This heterogeneity is also observable in the tissues sampled, **as well as the in the ways samples were conserved (i.e. time between sampling and freezing)**. We thus observed a sampling bias toward some combination of the evaluated factors (i.e. adults were samples only from muscles and internal organ). This imbalance of the data limits our ability to disentangle the effect of factors such as tissue, age or sex.”

Sequencing biases might also have consequences on our results, but we think our approach managed to overcome most of the biases.

All the individuals were sequenced to have a genome-wide coverage of around 15X (see Machado et *al.* 2021, Cumer et *al.* 2022, Cumer et *al.* 2023 and Machado et *al.* 2022 for details). However, we do observe inter-individual variation in the number of sequenced reads attributed to the mitochondrial genome, with a median individual coverage varying between 35 X and 24269 X, and a median of 606 X. Also, we did observe a link between the overall coverage and the tissue used for extraction, with higher coverage in muscles compared to other tissues (see attached figure). We think this might be due to the higher number of mitochondria in muscles, hence a higher proportion of mtDNA compared to genomic DNA.

We could also observe a positive relation between the median coverage of the coding region and the coverage of the repeated region. This is expected and shows that the repeated reads come from the repeated region of the mitochondrial genome.

As explained line 127, “The number of copies for each repeat was then computed by comparing the mean coverage of the repeated region (between 16750 and 16900 bp for the long repeat and between 17005 and 17025 bp for the short repeat) compared to the median coverage of the whole mitochondrial genome (Sup. Fig. 2).” After this normalization, the number of short and long repeats negatively correlate with the median coverage, meaning that less covered mitochondrial genomes seems to have more repeats.

To account for the potential effect of the total coverage of the mitochondrial genome, we re-ran the models including the median coverage of the coding region. The new version of the manuscript extensively describes the results. In brief, including this factor in our models did not change the results. For the *long* repeat, the median coverage was excluded from the model since models without this factor had a better fit. For the *short* repeat, the median coverage was kept in the final model but did not have a significant contribution (p-value = 0.17).

Regarding the nuclear mitochondrial DNA (numt), no numts were ever reported in the barn owl genome and a BLAST of the mitochondrial genome on the reference genome confirmed this result (data not sown). We cannot exclude the presence of unassembled numts in the genome, however, with a mean genomic coverage of 15X, the contribution of a hypothetic numt to our estimation of number of copies in the mitochondrial genome seems marginal.

Finally, we agree with the reviewer that a carefully sampled design may be needed to formally test our hypotheses, something we mention line 349:

“In the future, it will be informative to track the same individuals across time and sample different tissues (such as blood or feathers). This would allow to describe the variation of the repetition within different tissues of the same individuals through time”

Minor comments :

‘widespread’ may be more correct than ‘widely spread’

[R] changed, see line 69.
