# Supplementary material for: Characterization of the diversity of barn owl’s mitochondrial genome reveals high copy number variations in the control region
Source: PLoS One. 2024 Jan 25;19(1):e0295595. doi: 10.1371/journal.pone.0295595 (PMC10810427; doi:10.1371/journal.pone.0295595)
Supplement: Attachment — Submitted filename: 0_Tyto_mtDNA_AnswerReviewer.docx [file pone.0295595.s013.docx]

PONE-D-23-20889

Characterization of the diversity of barn owl’s mitochondrial genome reveals high copy number variations in the control region.

PLOS ONE

----------------------------------------------------------------------------------------------------------------------------------------

Dear Dr. Cumer,

Thank you for submitting your manuscript to PLOS ONE. After careful consideration, we feel that it has merit but does not fully meet PLOS ONE’s publication criteria as it currently stands. Therefore, we invite you to submit a revised version of the manuscript that addresses the points raised during the review process.

Please submit your revised manuscript by Dec 18 2023 11:59PM. If you will need more time than this to complete your revisions, please reply to this message or contact the journal office at [plosone@plos.org](mailto:plosone@plos.org). When you're ready to submit your revision, log on to <https://www.editorialmanager.com/pone/>and select the 'Submissions Needing Revision' folder to locate your manuscript file.

Please include the following items when submitting your revised manuscript:

- A rebuttal letter that responds to each point raised by the academic editor and reviewer(s). You should upload this letter as a separate file labeled 'Response to Reviewers'.
- A marked-up copy of your manuscript that highlights changes made to the original version. You should upload this as a separate file labeled 'Revised Manuscript with Track Changes'.
- An unmarked version of your revised paper without tracked changes. You should upload this as a separate file labeled 'Manuscript'.

If you would like to make changes to your financial disclosure, please include your updated statement in your cover letter. Guidelines for resubmitting your figure files are available below the reviewer comments at the end of this letter.

If applicable, we recommend that you deposit your laboratory protocols in [protocols.io](http://protocols.io) to enhance the reproducibility of your results. [Protocols.io](http://Protocols.io) assigns your protocol its own identifier (DOI) so that it can be cited independently in the future. For instructions see: [https://journals.plos.org/plosone/s/submission-guidelines#loc-laboratory-protocols](https://journals.plos.org/plosone/s/submission-guidelines#loc-laboratory-protocols). Additionally, PLOS ONE offers an option for publishing peer-reviewed Lab Protocol articles, which describe protocols hosted on [protocols.io](http://protocols.io). Read more information on sharing protocols at <https://plos.org/protocols?utm_medium=editorial-email&utm_source=authorletters&utm_campaign=protocols>.

We look forward to receiving your revised manuscript.

Kind regards,

Sven Winter
Academic Editor
PLOS ONE

Journal Requirements:

Please review your reference list to ensure that it is complete and correct. If you have cited papers that have been retracted, please include the rationale for doing so in the manuscript text, or remove these references and replace them with relevant current references. Any changes to the reference list should be mentioned in the rebuttal letter that accompanies your revised manuscript. If you need to cite a retracted article, indicate the article’s retracted status in the References list and also include a citation and full reference for the retraction notice.

**Additional Editor Comments:**

The revised manuscript did greatly improve and the reviewer, who has seen the revision, seems satisfied with the authors replies. I agree with the reviewer that all generated data (rawdata and assembled data) needs to be submitted to a public database before the manuscript can be accepted. Please also ensure, that all figures are submitted in a high enough resolution for publishing. 

[R] We have now deposited all the data used for this publication on public databases:
- Raw reads can be found via the NCBI website. The bioprojects of the different individuals can be found in table S2.
- The mitochondrial genome assembled for each individuals, the table of the coverage along the MT genome used to estimate the number of copies in each individual genome, the alignment used to build the tree in Figure 2, as well as the reference genome used in this study can now be found here :
<https://github.com/cumtr/Serrano_et_al_2023_Diversity_mitochondrial_genome_Barn_Owl>

We have now specified this information line 122 of the manuscript:

“Individual sequences can be downloaded at <https://github.com/cumtr/Serrano_et_al_2023_Diversity_mitochondrial_genome_Barn_Owl>”

Reviewers' comments:

Reviewer's Responses to Questions

**Comments to the Author**

1. If the authors have adequately addressed your comments raised in a previous round of review and you feel that this manuscript is now acceptable for publication, you may indicate that here to bypass the “Comments to the Author” section, enter your conflict of interest statement in the “Confidential to Editor” section, and submit your "Accept" recommendation.

Reviewer #2: All comments have been addressed

2. Is the manuscript technically sound, and do the data support the conclusions?

The manuscript must describe a technically sound piece of scientific research with data that supports the conclusions. Experiments must have been conducted rigorously, with appropriate controls, replication, and sample sizes. The conclusions must be drawn appropriately based on the data presented.

Reviewer #2: Yes

3. Has the statistical analysis been performed appropriately and rigorously?

Reviewer #2: Yes

4. Have the authors made all data underlying the findings in their manuscript fully available?

The [PLOS Data policy](http://www.plosone.org/static/policies.action#sharing) requires authors to make all data underlying the findings described in their manuscript fully available without restriction, with rare exception (please refer to the Data Availability Statement in the manuscript PDF file). The data should be provided as part of the manuscript or its supporting information, or deposited to a public repository. For example, in addition to summary statistics, the data points behind means, medians and variance measures should be available. If there are restrictions on publicly sharing data—e.g. participant privacy or use of data from a third party—those must be specified.

Reviewer #2: No

[R] All the data used in this paper are now available online. See our comment above for details.

5. Is the manuscript presented in an intelligible fashion and written in standard English?

PLOS ONE does not copyedit accepted manuscripts, so the language in submitted articles must be clear, correct, and unambiguous. Any typographical or grammatical errors should be corrected at revision, so please note any specific errors here.

Reviewer #2: Yes

6. Review Comments to the Author

Please use the space provided to explain your answers to the questions above. You may also include additional comments for the author, including concerns about dual publication, research ethics, or publication ethics. (Please upload your review as an attachment if it exceeds 20,000 characters)

Reviewer #2: The authors may need to submit all the assembled mitochondrial genomes to Genbank or EMBL to make the data avalaible to researchers interesting in replicating the analyses performed in the analyses. Figure is not available in the revised version but the version in the first version was very blurry.
Apart from this all comments that could be addressed were addressed properly

[R] All the data used in this paper are now available online. See our comment above for details.
We have also uploaded figures in high quality for publication.
